# Effects of different sperm sources on the clinical outcomes of *in vitro* oocyte maturation cycles combined with intracytoplasmic sperm injection

**DOI:** 10.3389/fendo.2023.1115210

**Published:** 2023-02-20

**Authors:** Jianhua Li, Jing Chen, Shuang Tian, Tingting Jiao, Jianye Wang, Yan Wei, Yanbin Cheng, Ye Xu, Ri-Cheng Chian, Youzhu Li, Shuiwen Zhang

**Affiliations:** ^1^ Reproductive Medical Center, Department of Obstetrics and Gynecology, Seventh Medical Center of PLA General Hospital, Beijing, China; ^2^ Reproductive Medicine Center, The First Affiliated Hospital of Xiamen University, Xiamen, China; ^3^ Center for Reproductive Medicine, Shanghai Tenth People’s Hospital of Tongji University, Shanghai, China

**Keywords:** percutaneous epididymal sperm aspiration (PESA), testicular sperm aspiration (TESA), ejaculated sperm, *in vitro* maturation (IVM), intracytoplasmic sperm injection (ICSI)

## Abstract

**Objectives:**

To evaluate the embryonic developments and clinical outcomes of different sperm sources with cycles of intracytoplasmic sperm injection (ICSI) and *in vitro* maturation (IVM).

**Methods:**

This retrospective study was approved by the hospital ethics committee and conducted in the hospital *in vitro* fertilization (IVF) clinic. From January 2005 to December 2018, 239 infertile couples underwent IVM–ICSI cycles and were divided into three groups according to different sperm sources. Group 1 comprised patients with percutaneous epididymal sperm aspiration (PESA; n = 62, 62 cycles), group 2 comprised patients with testicular sperm aspiration (TESA; n = 51, 51 cycles), and group 3 comprised patients with ejaculated sperm (n = 126, 126 cycles). We calculated the following outcomes: 1) outcomes per IVM–ICSI cycle: fertilization rate, cleavage rate, and embryo quality; 2) outcomes per embryo transfer cycle: endometrial thickness, implantation rate, biochemical pregnancy rate, clinical pregnancy rate, and live birth rate.

**Results:**

There was no difference in basic characteristics among the three groups, such as the female partner’s age, basal follicle-stimulating hormone (FSH), basal luteinizing hormone (LH), and antral follicle count (p > 0.1). There were no statistically significant differences according to the IVM–ICSI cycle among the three groups in fertilization rate, cleavage rate, and rate of good-quality embryos (p > 0.05). The results were similar among cycles regarding the number of transfer embryos and endometrial thickness per embryo transfer cycle among the three groups (p > 0.05). There were also similar clinical outcomes per embryo transfer cycle among the three groups, such as the biochemical pregnancy rate, clinical pregnancy rate, and live birth rate (p > 0.05).

**Conclusions:**

Different sperm sources, percutaneous epididymal sperm aspiration, testicular sperm aspiration, and ejaculated sperm, do not affect the embryo and clinical outcomes after IVM–ICSI cycles.

## 1 Introduction

Azoospermia refers to the absence of sperm in semen after three consecutive semen examinations and accounts for 15% of cases of male infertility (1). Sperm is initially generated in the seminiferous tubules of the testis, collected through these tubules, and temporarily stored in the epididymis. During ejaculation, sperm is discharged from the body through the vas deferens, ejaculatory ducts, and urethra. Azoospermia can be divided into obstructive azoospermia (OA) and non-obstructive azoospermia (NOA) according to the etiology. Non-obstructive azoospermia is a severe impairment or loss of testicular spermatogenesis function, while obstructive azoospermia refers to when the sperm production of the testicle is normal but the delivery pipeline (the epididymis or vas deferens) is blocked or absent.

Intracytoplasmic sperm injection (ICSI) can assist fertility by directly injecting sperm into an oocyte. This method has been the mainstream technique to help azoospermia patients produce genetic offspring (1). Percutaneous epididymal sperm aspiration (PESA) and testicular sperm aspiration (TESA) are common surgical methods for azoospermia patients to retrieve sperm for further ICSI (1). Recent research shows that sperm may carry genetic information that affects the development of offspring and control the early development of embryos (2). Sperm first shows two peaks of piRNA production in the testis and then experiences a large loss of piRNAs and an increase of tRNA fragments in the process of post-testicular maturation. Finally, sperm matures in the cauda (tail) epididymis with stronger forward movement ability and fertilization ability (3).

Small RNAs and microRNA changes mainly occur from the caput to the cauda of the epididymis. The sperm in the caput epididymis carries higher loads of tRF-Glu-CTC and tRF-Gly-GCC than sperm in the cauda epididymis, while sperm in the cauda epididymis has ten times more tRF-Val-CAC than sperm in the caput epididymis. MicroRNAs also dramatically vary during sperm maturation. For example, sperm in the cauda epididymis has a higher level of miR-17-92 oncomir clusters than sperm in the caput epididymis (4).

*In vitro* maturation (IVM) of the immature oocyte is an alternative to controlled ovarian hyperstimulation (COH). IVM can improve the utilization rate of oocytes, reduce the risk of ovarian hyperstimulation, and reduce cost (5). Due to the limited application of IVM in *in vitro* fertilization (IVF) laboratories, although there is no contraindication for IVM, it is still very difficult for couples with azoospermia and other types of male infertility to choose TESA–IVM or PESA–IVM. However, it is easier for such couples to make IVM decisions due to the risk of ovarian hyperstimulation syndrome (OHSS) and repeat IVF failure cycles, as well as the advantages of IVM, such as low cost, no OHSS risk, and repeatability in a short period after the failure of a traditional IVF cycle.

The maturity of sperm obtained from the testis, the epididymis, or ejaculation is different, and further clarification is needed in regard to whether these differences ultimately affect fertilization, embryo development, and clinical pregnancy. There is limited research on TESA–IVM and PESA–IVM cycles, and only a limited number of studies have reported on IVM oocytes fertilized by sperm from male patients with azoospermia in the ICSI cycle (6, 7). It is still debatable whether the source of sperm affects outcomes in the ICSI cycle or the IVM–ICSI cycle (1, 8). Therefore, we used different sperm sources (percutaneous epididymal sperm aspiration, testicular sperm aspiration, and ejaculated sperm) to analyze outcomes after ICSI in IVM cycles.

## 2 Materials and methods

### 2.1 Design and patients

This retrospective study was approved by the ethics committee at the Seventh Medical Centre of PLA General Hospital IVF clinic, where the study was conducted. Patients who underwent an IVM–ICSI cycle from January 2005 to December 2018 were included. The inclusion criteria were a normal karyotype of the female, normal uterine cavity and bilateral ovaries, fallopian tubes free of hydrosalpinx, and more than seven antral follicles. Azoospermia was diagnosed if no sperm was found in the male partner in three semen examinations and microscopic examinations after centrifugation and sedimentation. For a male with azoospermia, if the karyotype and azoospermic factor gene (AZF) were normal, on the day of oocyte retrieval, an andrologist checked the azoospermia patient and determined whether there was obstructive azoospermia (OA) or non-obstructive azoospermia (NOA) according to the history of obstruction, testicular volume, and hormone level.

All enrolled cases were undergoing an IVM–ICSI cycle for the first time. On the day of the female partner’s oocyte retrieval, the patients were classified by temporary semen extraction. OA patients whose sperm was aspirated from the corpus of the epididymis by PESA were classified as the PESA group (group 1). If PESA did not obtain sperm, then TESA was performed to extract sperm, and such patients were classified as the TESA group. Patients with NOA for whom TESA was performed directly were also considered as part of the TESA group (group 2). Group 3 was made up of male patients whose semen could be obtained directly by ejaculation. There were 26 cases in the PESA group and 31 cases in the TESA group, and all patients gave written informed consent.

### 2.2 Oocyte collection and IVM

On day 3 of menstruation, the number of follicles was monitored by vaginal ultrasound, excluding cyst formation. The monitoring was repeated 7–9 days after menstruation. When the dominant follicles reached 12–14 mm or the endometrial thickness was ≥6 mm, human chorionic gonadotropin (hCG) was triggered (10,000 IU), and then oocytes were collected 36 h later. The maturation of oocytes was evaluated under an anatomical microscope, and immature oocytes in metaphase I (MI) and germinal vesicle (GV) stages were matured in the IVM medium *in vitro* (5).

### 2.3 Sperm preparation and ICSI

Sperm was prepared using ejaculated sperm, caput epididymal sperm obtained by PESA, or testicular sperm obtained by TESA. For PESA, after applying local anesthesia, a fine needle was used to puncture the epididymal head with a 1-ml sperm-washing syringe. The sperm was aspirated and analyzed under an optical microscope. If no sperm was recovered, TESA was immediately performed by percutaneous puncture of testicular tissue with a needle, extraction of a convoluted seminiferous tubule with fiber tweezers, and microscopic examination of sufficient sperm. If necessary, the operation was repeated until there was enough sperm for ICSI. For patients with NOA, TESA was performed directly. Then, the *in vitro* matured oocytes were inseminated with sperm by ICSI.

### 2.4 Embryo culture and transfer

At 17 to 19 h after ICSI, fertilization was evaluated by the appearance of two pronuclei (2PN). Embryos with six cells on day 3 after ICSI and <20% fragments with regular morphology were evaluated as good-quality embryos. On day 3 after ICSI, the embryo was transferred. This study only counted the pregnancy data of fresh embryo transfers. Biochemical pregnancy was determined by the serum hCG on day 14 after embryo transfer. Clinical pregnancy was determined by the presence of an intrauterine gestational sac by ultrasound after 14 days of biochemical pregnancy.

### 2.5 Statistical analysis

SPSS 20.0 (IBM, Armonk, New York, USA) was used for statistical analysis. The Kruskal–Wallis test was used to statistically analyze data. P-values of less than 0.05 were considered statistically significant, and the results were expressed as the mean standard error.

## 3 Results

From January 2005 to December 2018, 239 infertile couples underwent ICSI–IVM cycles and were divided into group 1 (PESA, n = 62, 62 cycles), group 2 (TESA, n = 51, 51 cycles), and group 3 (n = 126, 126 cycles). There was no difference in basic characteristics among the three groups, such as the female partner’s age, basal follicle-stimulating hormone (FSH), basal luteinizing hormone (LH), and antral follicle count (p > 0.1, **Table 1**). There was no statistically significant difference per ICSI–IVM cycle among the three groups in fertilization rate, cleavage rate, and rate of good-quality embryos (p > 0.05, **Table 1**).

**Table 1:** Patient characteristics and embryology information of different sperm sources in IVM–ICSI cycle.

Variable	Sperm origin			p-Value
	PESA	TESA	Ejaculated	
No. of IVM–ICSI cycles (no. of patients)	62	51	126	
Female partner’s age (years)	28.76 ± 4.19	29.61 ± 3.92	29.85 ± 2.92	0.15
Basal FSH (IU/ml)	6.25 ± 3.35	6.05 ± 2.08	5.78 ± 1.89	0.66
Basal LH (IU/ml)	3.25 ± 2.28	2.89 ± 1.74	2.73 ± 2.00	0.25
Antral follicle count	14.73 ± 5.84	15.39 ± 6.01	14.11 ± 4.37	0.766
Fertilization rate/per oocyte for ICSI	82.44 ± 16.49	79.64 ± 22.72	86.66 ± 13.47	0.193
Cleavage rate/per fertility	86.23 ± 19.49	82.65 ± 25.02	82.80 ± 18.83	0.313
No. of embryo/cycle	2.74 ± 1.11	3.22 ± 1.73	2.97 ± 0.77	0.082
good quality embryo rate/cycle	16.67 ± 28.94	21.78 ± 26.88	21.16 ± 29.24	0.249

Numbers are mean ± SD unless otherwise indicated.

No., number; IVM, in vitro maturation; ICSI, intracytoplasmic sperm injection; PESA, percutaneous epididymal sperm aspiration; TESA, testicular sperm aspiration; FSH, follicle-stimulating hormone; LH, luteinizing hormone.

There were two cases in group 2 with no embryos available, so the total number of embryo transfer cycles in that group was 49. The total number of transplants in group 1 was 62, and that in group 3 was 126. The results were similar among cycles regarding the number of transfer embryos and endometrial thickness per embryo transfer cycle among the three groups (p > 0.05, **Table 2**). There were also similar clinical outcomes per embryo transfer cycle among the three groups, such as the biochemical pregnancy rate, clinical pregnancy rate, and live birth rate (p > 0.05, **Table 2**).

**Table 2:** Comparison of clinical and obstetric outcomes based on different sperm sources in IVM–ICSI cycle.

Variable	Sperm origin			p-Value
	PESA	TESA	Ejaculated	
No. of embryo transfer cycle	62	49	126	
No. of embryo transfer/cycle	2.50 ± 0.62	2.55 ± 0.58	2.70 ± 0.46	0.084
Endometrial thickness (mm)	7.27 ± 1.15	7.24 ± 1.44	7.45 ± 1.39	0.53
Implantation rate/cycle	18.01 ± 25.83	24.49 ± 35.69	21.43 ± 31.96	0.819
Biochemical pregnancy rate/cycle	28 (45.16%)	23 (46.93%)	58 (46.03%)	0.983
Miscarriage/cycle	3	2	8	
Clinical pregnancy/cycle	24 (38.71%)	20 (40.81%)	50 (39.68%)	0.975
Live birth (ongoing pregnancy)/cycle	22 (35.48%)	18 (36.73%)	45 (35.71%)	0.989
Singleton/cycle	18 (29.03%)	14 (28.57%)	31 (24.60%)	
Twins/cycle	4 (6.45%)	4 (8.16%)	14 (11.11%)	
Birthweight (g)				
Singleton	3,377 ± 465	3,565 ± 353	3,325 ± 263	0.101
Twins	2,341 ± 420	2,655 ± 250	2,450 ± 412	0.246

Numbers are mean ± SD unless otherwise indicated.

No.: number; IVM, in vitro maturation; ICSI, intracytoplasmic sperm injection; PESA, percutaneous epididymal sperm aspiration; TESA, testicular sperm aspiration.

## 4 Discussion

When couples are infertile due to male factors, such as azoospermia, they often question the impact of surgical sperm retrieval on the pregnancy rate and birth rate. It may also present significant challenges for them to choose a method of assisted pregnancy, and some people will even give up the chance to have genetic offspring because of too much anxiety (9). Due to the progress of science and technology, surgical sperm retrieval has enabled more and more men with azoospermia to have offspring. Previous research showed that ICSI using ejaculated or surgically retrieved sperm produced similar fertilization rates and pregnancy rates (10). However, there are few reports of successful pregnancies using surgically retrieved spermatozoa for ICSI cycles to fertilize IVM immature oocytes (6, 7).

*In vitro* maturation is a good choice for infertile women with normal ovarian function because they have a certain number of antral follicles in their ovaries, which can reduce the risk of the ovarian hyperstimulation syndrome. In addition, *in vitro* maturation can significantly reduce the required dosage of FSH and the cost of treatment (11). Due to the time efficiency, cost efficiency, and convenience of IVM, it has become a mature technique in our laboratory. The clinical pregnancy rate of IVM in our center is 30%–40% (12, 13), and the cumulative live birth rate is 66%–69% (12). These results have driven men who suffer from azoospermia to be inclined to surgical sperm retrieval combined with IVM. However, there are few reports on its clinical outcome (14, 15). In this study, we used different sperm sources to analyze the outcomes of ICSI using surgical sperm retrieval combined with IVM cycles, which may help patients make informed decisions.

Conine et al. inferred that caput sperm cannot undergo full-term maturation in FVB mice. They aspirated sperm from the caput and cauda of the epididymis in FVB mice and used them for fertilization by ICSI, and they found that multiple genetic materials (RNA) were highly expressed in zygotes fertilized by caput sperm, but the implantation rate was lower than in the cauda sperm group (2). Zygote fertilized by caput sperm began from the four-cell stage and continued to the blastocyst stage of development and expressed about 50 genes mainly encoding the regulation of RNA-binding protein and chromatin-related genes. In addition, purifying the small RNAs from cauda sperm and then injecting them into embryos formed by caput sperm can remedy the defects of the early embryo gene regulation and post-implantation development (2). However, other research showed that caput sperm is completely capable of full-term development in 129Sv/Ev, BDF1, C57BL/6NHsd, FVB, and CD1 mice (16–18). Our study also supports that no obvious differences were found among different sperm sources on fertilization rate and clinical pregnancy rate.

Some researchers found that compared with the sperm from TESA, the sperm from PESA is more likely to lead to fertilization and produce more usable blastocysts. However, its euploid rate is similar, and the pregnancy rate of the first embryo transfer cycle also has no significant difference when compared with the ejaculation group and TESA group (19). Multiple cumulative transplantation cycles were required to observe the clinical outcome. We have come to the same conclusion since sperm recovered by PESA and TESA had similar clinical pregnancy rates and birth rates, and there was no significant difference compared to the ejaculation group. Another study found that the rate of high-quality blastocysts in the TESA group (82.56%) was significantly higher than that in the PESA group (71.82%), but the embryo implantation rate, pregnancy rate, and miscarriage rate of spermatozoa from the PESA and TESA groups were similar during the ICSI cycle (1).

Sperm obtained by TESA is fresh sperm in testicular tissue, while sperm obtained by PESA is stored in the caput of the epididymis for a long time, resulting in high DNA fragmentation of the sperm, which ultimately affects the quality of embryos (20). Another analysis showed that the DNA fragmentation rate of testicular sperm was lower than that of ejaculatory sperm, and the live yield of TESA-ICSI was higher than that of ejaculatory ICSI (21). Although the embryo quality of the PESA group was slightly poor, the best embryo quality was routinely selected for transfer in the first transfer cycle, so there was no significant difference in the clinical pregnancy rate and abortion rate.

As the sperm obtained by PESA and TESA is processed in the laboratory, sperm with good shape and vitality are selected for ICSI, so as long as the woman’s age, endometrium, and other conditions are good, there is no significant impact on the pregnancy outcome (1). In addition, no abnormal increase of DNA was found in sperm cells of epididymis by using the TdT-mediated dUTP nick-end labeling assay, and ICSI combined with PESA and TESA did not increase the birth rate, malformation rate, and abortion rate (22). Based on the physiological and biochemical characteristics of testicular sperm, it is immature. However, they are completely adequate from the perspective of ICSI fertilization (23). Our research data support this, suggesting that the fertilization rate, pregnancy rate, and birth rate of testicular sperm are not significantly different from those of epididymal sperm and ejaculatory sperm.

One limitation of our study is that the sample size was not large enough. Also, we did not carry out euploid analysis on embryos, and we were unable to judge the quality of embryos at the genetic level. Finally, we only followed up on the birth of children but did not examine the deformity rate of children over the long term. Therefore, more comprehensive and larger samples and longer follow-up periods are needed to confirm the results.

In conclusion, infertile couples with azoospermia alone can achieve satisfactory outcomes regardless of whether the sperm is from the testis or epididymis. There were no significant differences in fertilization rate, clinical pregnancy rate, and birth rate in the IVM cycle between these types of sperm, and the rates were also equivalent to those of the ejaculated sperm group. In clinical practice, most doctors will choose PESA due to its advantages, such as less trauma, less bleeding, rapid recovery, and fewer complications (24). Therefore, for men with azoospermia, the source of sperm has no significant impact on the IVM cycle, and the selection of TESA or PESA should be considered comprehensively. The advantages and disadvantages of multiple factors should be weighed to determine the actual prognosis to make the most appropriate and reasonable treatment plan.

## Data availability statement

The original contributions presented in the study are included in the article/Supplementary Material. Further inquiries can be directed to the corresponding authors.

## Ethics statement 

This study was approved by the Ethics Committee of the Seventh Medical Center of PLA General Hospital. The patients/participants provided their written informed consent to participate in this study.

## Author contributions

SZ, YL, and R-CC conceived and designed the study. JL, JC, JW, ST, and YX performed the experiments and analyzed the data. TJ, YW, and YC collected the data. JL and JC wrote and revised the manuscript. All authors contributed to the article and approved the submitted version.
